# Genetic Basis of Brugada Syndrome

**DOI:** 10.3390/biomedicines13071740

**Published:** 2025-07-16

**Authors:** Xianghuan Xie, Yanghui Chen, Zhiqiang Li, Yang Sun, Guangzhi Chen

**Affiliations:** 1 Hubei Key Laboratory of Genetics and Molecular Mechanisms of Cardiological Disorders, Division of Cardiology, Department of Internal Medicine, Tongji Hospital, Tongji Medical College, Huazhong University of Science and Technology, 1095# Jiefang Ave., Wuhan 430030, China; m202476596@hust.edu.cn (X.X.);; 2 Department of Cell Biology, SUNY Downstate Health Sciences University, Brooklyn, NY 11203, USA; zhiqiang.li@downstate.edu

**Keywords:** Brugada syndrome, Brugada, genetics, genes

## Abstract

Brugada syndrome is a rare inherited heart disease characterized by ventricular arrhythmias and characteristic ST segment elevation, which increases the risk of sudden death. Studies have shown that the pathogenesis of this disease involves a variety of gene mutations, including abnormal functions of sodium, calcium, and potassium ion channels, resulting in cardiac electrophysiological disorders. These variants affect excitability and conduction of cardiomyocytes, thereby increasing the susceptibility to ventricular arrhythmias and sudden death. However, many genetic variants remain of uncertain significance or are insufficiently characterized, necessitating further investigation. This review summarizes the genetic variants associated with Brugada syndrome and discusses their potential implications for improving diagnosis and therapeutic approaches.

## 1. Introduction

Brugada syndrome (BrS) is a rare genetic disorder characterized by specific ECG findings, such as ST segment elevation in the right precordial leads (V1–V3) and increased risk of sudden cardiac death because of ventricular arrhythmias [1]. It is primarily caused by mutations in genes encoding cardiac ion channels or their regulatory proteins, leading to abnormal electrical activity in the heart. BrS mainly affects young men of Southeast Asian and Japanese descent [2]. While BrS is predominantly diagnosed in adults, recent studies suggest that the condition is also present in the pediatric population, albeit underrepresented in clinical research. Despite its rarity, the incidence of fatal arrhythmias in children with BrS is estimated to be around 10% [3]. It is electrocardiographically defined as polymorphic ventricular tachycardia, which may eventually progress to ventricular fibrillation. This fibrillation causes the heart to beat abnormally rapidly and dangerously, eventually leading to sudden cardiac arrest [4]. Patients with BrS may experience episodes of unexplained palpitations or fainting. Diagnosis is usually based on specific tests, including electrocardiography [5]. Treatment may include administering drugs to stabilize heart rhythm, implanting defibrillators capable of delivering electrical impulses in potentially fatal arrhythmia, and introducing lifestyle changes to reduce triggering factors, such as fever or certain pharmacological agents [6].

**Figure 1:**
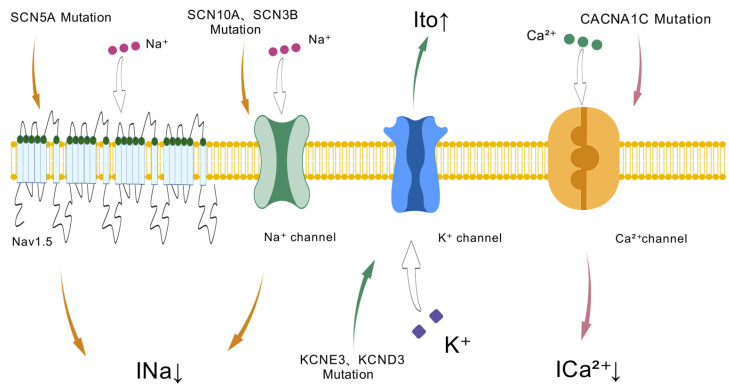
Ion channel dysfunction in Brugada syndrome: Genetic mutations in sodium (SCN5A, SCN10A, SCN3B), potassium (KCNE3, KCND3), and calcium (CACNA1C) channels lead to altered ion currents (INa, Ito, Ica^2+^), contributing to the electrophysiological abnormalities seen in Brugada syndrome.

Evidence suggests that BrS may be oligogenic or polygenic in some cases. Mutations in multiple genes play crucial roles in cardiac ion channels and their regulatory proteins [7]. (Figure 1) In this comprehensive review, we aim to provide an in-depth understanding of the genetic mutations associated with BrS, delving into their importance in cardiac ion channel malfunction, arrhythmia genesis, and clinical manifestations [8]. By disentangling the intricate genetic underpinnings of BrS, we aspire to facilitate the development of personalized diagnostic, prognostic, and therapeutic strategies for this potentially fatal condition.

## 2. Genetic Basis

In the context of genetically determined BrS, mutations within the *SCN5A* gene located on chromosome 3p21-23 contribute to 20–30% of BrS cases within the genotyped family cohorts [9]. Recently, mutations in other genes have been associated with the clinical phenotypes of BrS. In this review, we summarize the candidate and well-established genes associated with BrS (Table 1). This includes mutations that reduce the sodium channel current function, mutations that enhance the potassium channel current function, mutations that decrease the calcium channel current function, and other potential genes that may be involved [10].

## 3. Mutations Causing a Loss-of-Function of Sodium Channel Current

### 3.1. SCN5A

In 1998, Chen et al. identified the primary genetic alteration linked to BrS in the *SCN5A* gene, which encodes the Na_V_1.5 sodium channel crucial for cardiac action potentials [11]. Mutations in *SCN5A* are present in approximately 75% of genotype-positive cases and 11–28% of all patients with BrS, leading to reduced sodium current and conduction anomalies that increase the risk of arrhythmia [11,12,13]. Several investigations have indicated that *SCN5A* mutations cause a reduction in sodium current, alter channel gating, and impede channel trafficking to the cell membrane, ultimately leading to conduction anomalies and an increased risk of arrhythmias [7]. Different mutations in *SCN5A*, which is associated with BrS, can generate varying degrees of the cardiac sodium current (INa) decline and possibly influence the clinical severity of its manifestations [14]. A multicenter study involving more than 2000 affected individuals revealed 293 distinct *SCN5A* variants, with the majority confined to single cases. This highlights the heterogeneity of the disease and the complexity of definitively labeling all variants as pathogenic [15]. Recent studies have increasingly utilized cardiomyocytes derived from induced pluripotent stem cells (iPSC–CMs) as experimental models. For example, the SCN5A p.C335R variant has been shown to cause a loss of peak sodium current (INa) function in patients with cardiac conduction diseases. Testing how specific SCN5A variants affect INa by transiently transfecting HEK-293 cells or similar model systems remains a commonly used method. Nevertheless, these models may not fully reproduce the complex electrophysiological behavior of cardiomyocytes in vivo and thus have limitations that require careful consideration. Furthermore, a comprehensive study by Kroncke et al., which analyzed all SCN5A variants documented in individuals with Brugada syndrome (BrS) or listed in the Genome Aggregation Database, identified at least 1712 distinct variants. Their analyses confirmed that a reduction in peak current is the most reliable predictor of an unfavorable clinical outcome [16].

Furthermore, disturbances in intracellular calcium regulation in cardiac cells may result from *SCN5A* mutations. A delicate interaction between sodium and calcium channels is essential for proper cardiac function, and impairments in calcium regulation play a role in the development of arrhythmias in BrS. Comprehending these underlying mechanisms is the key to unraveling the pathophysiology of this condition and advancing precise therapies [17].

In terms of risk stratification, recent studies have solidified the role of SCN5A mutations in predicting arrhythmic events in BrS patients. For example, a 2016 study by Antzelevitch et al. found a strong link between SCN5A mutations and an increased risk of spontaneous ventricular arrhythmias, including ventricular fibrillation (VF) and sudden cardiac death (SCD), emphasizing their role in identifying high-risk patients [18]. Additionally, a 2010 study by Nademanee et al. showed that SCN5A mutation carriers are more likely to experience early arrhythmic events, particularly those triggered by electrical stimulation. This supports the predictive value of SCN5A mutations for identifying individuals at risk for induced arrhythmias [19]. A 2024 study by Kato et al. highlighted that SCN5A loss-of-function mutations correlate with an increased risk of recurrent ventricular fibrillation during electrophysiological testing. This study solidifies the role of SCN5A mutations as not only a diagnostic marker but also a prognostic biomarker, useful for risk stratification and predicting the recurrence of ventricular arrhythmias in BrS patients [20].

Overall, the evidence highlights the crucial role of SCN5A mutations in BrS pathogenesis and their utility in risk assessment, although more research is needed to fully understand their mechanisms and therapeutic implications.

### 3.2. SCN10A

Na_V_1.8 (encoded by SCN10A) is a tetrodotoxin-resistant, voltage-gated sodium channel located adjacent to SCN5A on the human chromosome 3p21–22 [21]. Co-expression of wild-type SCN5A (which encodes the α-subunit of the cardiac sodium channel) with wild-type SCN10A enhances the sodium current (I_Na_) [22]. Conversely, the combination of wild-type SCN5A with mutated SCN10A has been associated with a reduction in INa, which may contribute to Brugada syndrome (BrS). But the evidence linking SCN10A to BrS remains controversial and may be confounded by linkage disequilibrium with SCN5A variants, given their close genomic proximity [18]. The identification of SCN10A as a predisposing gene in approximately 16.7% of BrS cases thus requires cautious interpretation [22].

Recent studies have suggested a potential association between other genes within this family and BrS. Mutations in the skeletal muscle sodium channel gene, *SCN4A*, may disrupt sodium and potassium currents in cardiac myocytes, potentially leading to the loss of an action potential dome in the right ventricular epicardium [23] and creating an arrhythmogenic substrate [24]. Similarly, alterations in SCN9A could influence NaV1.7 channel function in cardiac myocytes, potentially increasing susceptibility to BrS-related arrhythmias. However, these associations remain weak and speculative, and presenting them without qualification could be misleading. Further research is necessary to confirm any causative roles [25].

### 3.3. SCN1-4B

Variations in *SCN1-4B* have been linked to atrial fibrillation, cardiac conduction abnormalities, other arrhythmias, and BrS [26]. Research conducted by Watanabe and colleagues [27] identified variations in the *SCN1B* gene, which encodes the β1 and β1b subunits of Na_V_1.5. These mutations result in decreased I_Na_ currents. In a study involving three BrS patients without *SCN5A* mutations, Watanabe et al. observed that co-expression of mutant β1 or β1b subunits with Na_V_1.5 led to lower I_Na_ levels than those seen with wild-type subunits. These findings suggest a potential association of *SCN1B* with the pathogenesis of the disease. The transient outward potassium current (Ito), controlled by Kv4 potassium channels, causes the first phase of the heart’s action potential. Recent studies show that the Navβ1 subunit, when expressed with Kv4.3, changes Kv4.3’s behavior to better match natural Ito currents. Experiments also show Navβ1 physically binds to Kv4.2 and Kv4.3 in heart muscle cells. Reducing Navβ1 levels lowers the amounts of Nav1.5, Kv4.2, Kv4.3, and KChIP2, and decreases both INa and Ito currents. This suggests Nav1.5 and Ito channels form a complex through β1 subunits. Watanabe and colleagues found that the NaVβ1b subunit interacts structurally with NaV1.5 and Kv4.3, meaning SCN1B mutations can reduce INa and increase Ito. More Ito and less INa together may cause an outward current shift early in the action potential, especially in the right ventricle, where Ito is strong. This can lead to the heart rhythm problems seen in Brugada syndrome [28].

*SCN2B*, which encodes the β2 subunit of Na_V_1.5, forms co-immunoprecipitation complexes with Na_V_1.5 and colocalizes specifically at the intercalated disks within cardiac muscle cells. The manifestations of BrS may be attributed to uncommon genetic alterations within this specific gene. These variants have the potential to induce the BrS phenotype by causing a marked decrease in the density of the inward Na^+^ current as a result of ediminished cell surface expression of Na_V_1.5 [29].

Hu et al. demonstrated that *SCN3B*, which encodes the β3 subunit of Na_V_1.5, is involved in co-immunoprecipitation with Na_V_1.5. Mutations in *SCN3B* led to reduced I_Na_ density, hastened inactivation, and postponed channel reactivation. These effects are attributed to disruptions in intracellular trafficking and surface presentation of Na_V_1.5 [30].

It is important to note that the penetrance of variants in SCN1B, SCN2B, and SCN3B is limited, and their disease-causing roles remain unclear when studied in larger population cohorts. Many of these variants are rare, and current evidence does not fully establish a direct causal link between these mutations and Brugada syndrome or other arrhythmias. Therefore, further functional investigations and large-scale clinical studies are needed to clarify their pathogenic significance.

### 3.4. GPD1L

*GPD1L* is a crucial partner of *SCN5A*, and reduced *GPD1L* levels may affect Na_V_1.5 function. In 2007, Weiss et al. discovered a mutation (A280V) in *GPD1L* that led to an approximately 50% reduction in inward Na^+^ currents compared to those seen in wild-type *GPD1L* when expressed in HEK cells. Wild-type *GPD1L* is localized closer to the cell surface than the A280V variant, which causes a considerable 31 ± 5% decrease in the cell surface expression of *SCN5A* [31]. These findings highlight the effect of the A280V mutation on Na^+^ currents and *GPD1L* localization, which contribute to abnormal heart rhythms and BrS [32].

### 3.5. PKP2

Mutations in *PKP2* have been linked to arrhythmogenic cardiomyopathy and BrS, highlighting the relationship between desmosomal abnormalities and cardiac rhythm disturbances. In 2013, Marina et al. developed a new HL–1-derived cardiac cell line that endogenously expresses Na_V_1.5 but was deficient in PKP2 (PKP2–KD). Loss of PKP2 in these cells caused a decrease in the magnitude of I_Na_ and a decreased abundance of Na_V_1.5 at the site of cell contact. These findings suggest that the altered *PKP2* expression affects sodium and calcium channel localization, contributing to the arrhythmogenic traits of BrS [33]. Moreover, disruptions in *PKP2*-mediated signaling cascades have been associated with the development of conduction irregularities and ventricular arrhythmias, which are typically observed in patients with BrS [34].

### 3.6. HEY2

Brezzina et al. (2013) [35] reported that *Hey2* is associated with BrS. In *Hey2*-null mice, developmental abnormalities such as ventricular wall thinning and abnormal right ventricular morphology were observed. Loss of *Hey2* may disrupt the transmural expression gradient of Na_V_1.5 associated with BrS, and prolonged repolarization parameters suggest their regulatory role in repolarizing currents. Thus, *Hey2* is a crucial transcriptional regulator of cardiac electrical function and pathogenesis of BrS [35]. However, these findings are primarily based on mouse models, which may limit their direct applicability to human Brugada syndrome. Further studies in human tissues or clinical settings are needed to confirm the role of HEY2 in the disease.

### 3.7. Other Genes Related to Sodium Channel Current

The roles of ANK2, FGF12, RANGRF, and SLMAP in Brugada syndrome (BrS) remain speculative and are primarily supported by in vitro or animal model studies, with limited clinical validation in human cohorts.

*ANK2: ANK2* (ankyrin-B) is associated with BrS. Integration of heterozygous missense mutation G3157A into Na_V_1.5 inhibits Na_V_1.5 DII-DIII binding to ankyrin-G, leading to decreased Na_V_ channel surface expression and reduced inward Na+ current, potentially contributing to BrS [36].

*FGF12: FGF12* encodes fibroblast growth factor homologous factor 1 (FHF–1) [37]. The Q7R-*FGF12* mutant exhibited reduced binding to the Na_V_1.5 C terminus while maintaining normal interaction with junctophilin-2 [38]. In adult rat cardiac myocytes, the Q7R-*Fgf12* mutant decreased Na^+^ channel current density and availability, leading to reduced action potential amplitude without affecting Ca^2+^ channel function [39].

*RANGRF: RANGRF* encodes the MOG1 protein, a cofactor necessary for the proper functioning of Na_V_1.5. This gene plays a critical role in modulating the cardiac ion channel function and electrical conduction in the heart. MOG1 overexpression markedly increased whole-cell sodium currents in Na_V_1.5-expressing HEK-293 cells and cardiomyocytes [40]. While these results are promising, their translation to clinical BrS cases requires further investigation.

*SLMAP*: Mutations in *SLMAP* may contribute to BrS by altering the hNa_V_1.5 channel movement and increasing the risk of arrhythmia [41]. In 2012, Ishikawa et al. linked missense mutations in *SLMAP3* to Brugada patients in the Japanese population and provided evidence for a potential deficit in the trafficking of Na_V_1.5, a protein, to the surface membrane and channel activity in HEK-293 cells. Notably, missense mutations in the SLMAP3 isoform were identified in Japanese BrS patients, suggesting a population-specific association [42]. Elevated *SLMAP3* levels in the postnatal myocardium reduced Na_V_1.5 mRNA, leading to decreased channel protein in membranes and potentially prolonging the PR interval, affecting the cardiac function [43]. These findings highlight the importance of considering ethnic and population contexts when interpreting SLMAP’s role in BrS.

## 4. Mutations Causing a Gain-of-Function of Potassium Channel Currents

### 4.1. KCNE

Mutations in *KCNE3* can result in considerable changes in the function of associated potassium channels, ultimately affecting cardiac repolarization and contributing to the manifestation of BrS. In 2008, Delpon et al. detected a missense mutation (R99H) in *KCNE3* (MiRP2) in a proband. CHO–K1 cells were transfected with wild-type or mutant *KCNE3*, along with either wild-type *KCND3* or *KCNQ1*. Whole-cell patch-clamp studies were performed at 48 h post-transfection. Analysis of the interactions between K_v_4.3 and *KCNE3* was carried out using co-immunoprecipitation experiments on human atrial samples. Co-transfection of R99H *KCNE3* with *KCND3* resulted in a remarkable increase in Ito intensity compared to that of wild-type *KCNE3* with *KCND3* [44]. While these findings suggest that KCNE3 regulates Ito in the human heart, the direct role of KCNE3 mutations in BrS pathogenesis remains uncertain due to limited clinical evidence. Most data come from in vitro studies, and further patient-based research is needed to establish a definitive link.

Mutations in the *KCNE* gene family may contribute to BrS. *KCNE1* mutations associated with long QT syndrome have been found in BrS cases. These mutations affect the rapid delayed rectifier potassium current (IKr) and cardiac repolarization [45]. *KCNE2* mutations, such as D85N, can reduce the slow delayed rectifier potassium current (IKs), whereas variants such as I57T and M54T enhance the transient outward current, impacting I_to_ decay [46]. *KCNE4* considerably affects the *KCNQ1* current, potentially inhibiting channels without affecting surface expression, binding with calmodulin, or interacting with *KCNQ1* [47]. *KCNE5* mutations are associated with atrial fibrillation and BrS, which cause increased potassium currents and cardiac arrhythmias [47]. Specific mutations, such as *KCNE5*-L65F, shift the voltage independence of K_V_2.1-KCNE5 channels, while BrS-related mutations alter the voltage dependence of K_V_2.1. These findings suggest a mechanism for BrS linked to *KCNE5* mutations involving increased K_V_ currents [48].

### 4.2. KCND

In 2012, Crotti et al. identified *KCND3* mutations in two patients, affecting early repolarization through the I_to_ current in phase 1 of the cardiac action potential. This current exhibits a gradient across the ventricular myocardium, particularly in the right ventricular outflow tract. Disruption of the epicardial–endocardial current gradient, either by increased outward (I_to_, I_K-ATP_) or reduced inward (I_Na_, I_Ca_) currents, manifests as an ECG J-wave, creating an arrhythmogenic substrate (phase 2 reentry). The molecular basis of Ito centers around the K_V_4.3 channel encoded by *KCND3*, with its function regulated by *KCHIP2* [49]. *KCND3* mutations may contribute to BrS by heightening the I_to_ current, particularly in regions with high *KCND3* expression, such as the right ventricle, potentially leading to lethal arrhythmias. This emphasizes the crucial role of *KCND3* in the pathogenesis of BrS.

Historically, the K_V_4.2 channel encoded by *KCND2* was not considered crucial in the human myocardium. But recent studies on regional gene expression in healthy human hearts suggest that KCND2 may also be functionally important [50]. While this raises questions about its potential involvement in cardiac disease, further research is needed to determine whether KCND2 dysfunction directly contributes to arrhythmogenic conditions like BrS.

### 4.3. KCNJ8

Haissaguerre et al. first identified a *KCNJ8* mutation in a single case of early repolarization syndrome in 2009. Meideros-Domingo et al. discovered the S422L-*KCNJ8* mutation in early repolarization syndrome and BrS probands, establishing it as a missense mutation that was not found in 1200 reference control alleles [51]. Functional studies in COS-1 cells co-expressing KCNJ8-S422L and the ventricular SUR2A subunit showed a significant increase in KATP channel current compared to wild-type. This gain-of-function results from reduced sensitivity of Kir6.1-S422L channels to ATP inhibition, causing them to open more easily under normal conditions [52]. The main effect of the S422L mutation is enhanced epicardial KATP channel activity, which shortens the epicardial action potential duration. This creates a repolarization gradient across the heart wall, leading to the characteristic J-wave patterns seen in ERS and BrS patients with this mutation [53]. In summary, the mutation directly increases Kir6.1 channel function, while the resulting repolarization differences and arrhythmia risk are secondary effects. This explains how KCNJ8-S422L affects membrane excitability and contributes to arrhythmogenesis in J-wave syndromes [54]. However, conflicting data exist, and population studies are limited. The exact prevalence and penetrance of KCNJ8-S422L are still unclear, and some reports question the consistency of its functional effects. Therefore, while current evidence supports its role in J-wave syndromes, more research is needed to confirm its clinical significance and variability.

*KCNJ16* encodes the Kir5.1 subunit of potassium channels, which is crucial for maintaining cardiac electrical stability and repolarization. Although further research is required to clarify the specific role of *KCNJ16* in BrS, the current evidence suggests its potential importance in the pathogenesis of this disorder [55].

### 4.4. KCNH2

An investigation conducted in 2005 by Verkerk et al. involved voltage-clamp studies of transfected HEK-293 cells. These findings indicate a boost in I_Kr_ density, along with a shift toward negative voltage-dependent inactivation, ultimately leading to enhanced rectification. Through action potential clamp tests, augmented transient hERG peak currents (I_peak_) were observed during phase 0 and phase 1 of the ventricular action potential, particularly at shorter cycle lengths. Computational models further illustrated that heightened I_peak_ magnifies the risk of losing the action potential dome, a characteristic mainly evident in right ventricular subepicardial myocytes, and contributes considerably to the manifestation of the BrS phenotype [56]. In 2024, researchers conducted patch-clamp analysis of I_Kr_ reconstituted with a *KCNH2* mutation in Chinese hamster ovary cells and compared it with the wild-type phenotype. The results indicated that the *KCNH2* mutation did not affect the density of I_Kr_ but enhanced activation, impeded hERG channel inactivation, and increased the window current magnitude, suggesting a gain-of-function mutation [57]. The link between increased IKr and BrS is still debated. While increased IKr usually shortens the action potential and QT interval, BrS typically involves sodium channel loss-of-function and/or increased ITO. Therefore, KCNH2 gain-of-function mutations may play a secondary or complex role in BrS rather than being a primary cause. More research is needed to clarify their exact role and how they differ from effects in LQTS and other arrhythmias.

### 4.5. HCN4

The association between HCN4 dysfunction and cardiac arrhythmias was initially suggested by the identification of a splicing mutation in a suspected Brugada syndrome (BrS) patient (Ueda et al., 2009), with computational modeling indicating that the HCN4-mediated If current (characterized by mixed Na^+^/K^+^ permeability and incomplete deactivation) contributes to ventricular repolarization and may predispose to bradycardia-associated arrhythmias [58]. Recent studies of the V492F variant, which uniquely localizes adjacent to the sixth transmembrane segment (showing HCN4/HCN3-specific conservation) [59]. The mutations reveal near-complete loss of function in homomeric channels, which is partially rescued by wild-type subunits. Additionally, they cause a hyperpolarizing activation shift, which is typical of HCN4 loss-of-function. Despite the reduced current density, trafficking and cAMP sensitivity are preserved, although the underlying mechanism is unclear—it could be due to either decreased conductance or open probability. However, critical limitations include: absence of population/familial data for causal association, reliance on suspected rather than confirmed BrS diagnosis, and uncertainty whether observed electrophysiological changes represent primary pathogenic mechanisms or disease modifiers [60].

### 4.6. Other Genes Related to Potassium Channel Currents

*ABCC9*: ABCC9 encodes the SUR2A subunit of the KATP channel, which regulates cardiac repolarization via the IK-ATP current. Mutations like V734I cause gain-of-function effects by reducing ATP sensitivity, enhancing repolarization, and increasing arrhythmia risk. These mutations are linked to atrial fibrillation, cardiomyopathy, and myocardial infarction, and recent studies also implicate them in Brugada syndrome (BrS) and early repolarization syndrome (ERS) [61]. The role of ABCC9 in BrS is supported by pharmacological studies showing that KATP channel openers can induce BrS-like ECG patterns. Additionally, ABCC9 interacts with the Kv4.3 channel complex, which may modulate the Ito current. The direct relevance of these interactions to BrS remains unclear. Overall, whether ABCC9 mutations act as primary drivers or secondary modulators in BrS pathogenesis is yet to be determined [62].

*SEMA3A*: SEMA3A encodes semaphorin 3A, a protein known for guiding nerve growth and also important for the heart’s electrical system. Recent studies show that SEMA3A naturally inhibits the Kv4.3 potassium channel, which controls the fast transient outward potassium current (Ito) in heart cells. It reduces the Ito current without changing how much Kv4.3 is on the cell surface by directly interacting with the channel. In Brugada syndrome (BrS), increased Ito can cause dangerous heart rhythms. SEMA3A’s inhibition of Ito likely protects against BrS. But if mutations reduce SEMA3A’s function, this inhibition is lost, leading to higher Ito and increased risk of BrS. Therefore, SEMA3A is a potential BrS risk gene because its loss of function may cause excessive Ito. Further studies are needed to clarify the clinical impact of SEMA3A variants and their role in arrhythmia susceptibility [63].

Other genes such as *KCNK17* [64], *KCNB2*, *KCNQ1*, and *KCNT1* [55] may be associated with BrS, although the precise mechanisms linking these mutations to the syndrome are not fully understood.

## 5. Mutations Causing a Loss-of-Function of Calcium Channel Current

### 5.1. CACNA1C

Mutations in the *CACNA1C* gene have been implicated in various cardiac arrhythmia syndromes, including Brugada syndrome (BrS) and long QT syndrome type 8 (LQTS8, also known as Timothy syndrome). In the context of BrS, certain CACNA1C mutations cause a loss-of-function of the L-type calcium channel, resulting in reduced calcium influx during the plateau phase of the cardiac action potential. This reduction can lead to decreased action potential amplitude, altered repolarization heterogeneity, and an increased predisposition to ventricular arrhythmias such as ventricular tachycardia and ventricular fibrillation—hallmarks of BrS. Conversely, CACNA1C mutations associated with Timothy syndrome and LQTS8 typically produce a gain-of-function or altered channel gating, leading to prolonged action potentials and QT interval prolongation, which increase arrhythmia risk through different electrophysiological mechanisms [65]. In vitro studies using HEK-293 cells overexpressing two novel Cavα1.2 mutants demonstrated decreased Cavα1.2 protein expression at the plasma membrane, as evidenced by Western blot and cell surface biotinylation assays. Whole-cell patch-clamp recordings further showed significantly reduced calcium current density in mutant channels compared to wild-type controls, indicating impaired channel function [66]. Recent studies primarily focus on specific CACNA1C mutations in vitro studies, which may not fully reflect the complex in vivo cardiac environment or address long-term clinical implications. Additionally, it lacks patient-specific data and therapeutic insights, limiting the comprehensive understanding and application of these findings.

The CACNB2B gene encodes the beta-2 subunit of the cardiac L-type calcium channel, which is essential for proper electrical conduction. Recent patch-clamp studies in 2023 demonstrated a significant reduction in calcium current density in cells expressing mutant CACNB2B compared to wild-type, suggesting a loss-of-function effect. This loss is attributed to accelerated inactivation of the calcium current (ICa), leading to diminished calcium influx. However, these mechanistic claims remain preliminary without quantitative correlation to clinical phenotypes or arrhythmia risk. Moreover, data on mutation penetrance, familial segregation, and prevalence in BrS patients versus the general population are limited, underscoring the need for further genetic and epidemiological studies [67]. Further investigation is crucial to uncover the precise pathways by which mutations in *CACNB2B* induce BrS. 

Similarly, CACNA2D1, encoding the alpha-2/delta-1 subunit of the L-type calcium channel, plays a critical role in cardiac muscle function [68]. While mutations in CACNA2D1 have been associated with BrS, the precise molecular mechanisms remain unclear. Dysfunction of this subunit may impair calcium ion entry into cardiac myocytes, disrupting electrical signaling and predisposing to arrhythmogenesis. Yet, the clinical significance, mutation frequency, and inheritance patterns of CACNA2D1 variants in BrS require further investigation.

### 5.2. CASQ2

Calsequestrin 2 (*CASQ2*) is pivotal for the regulation of calcium release from the sarcoplasmic reticulum, and mutations can cause CPVT. However, recent studies have linked this condition to BrS. Regulation of calcium flux is essential for maintaining cardiac excitability and contractility. Mutations in *CASQ2* can disrupt the normal function of calsequestrin 2, leading to alterations in calcium handling within cardiac cells. This disruption can result in abnormalities in the electrical activity of the heart, affecting action potential duration and repolarization. These disruptions in calcium handling create a substrate for arrhythmias, thereby increasing the risk of ventricular arrhythmias and sudden cardiac arrest. These changes can predispose individuals to BrS-associated arrhythmias [69].

### 5.3. RYR2

Ryanodine receptor 2 (*RYR2*) is pivotal in the regulation of calcium release from the sarcoplasmic reticulum, and mutations in this gene can cause CPVT. Several studies have reported a correlation between *RYR2* mutations and BrS, highlighting the importance of calcium release channels in the pathophysiology of this syndrome. Additionally, the genetic variations in *RYR2* may influence the severity and clinical presentation of BrS in affected individuals. Mutations in RYR2 have been implicated in BrS pathogenesis. The ryanodine receptor is a large intracellular calcium release channel located in the sarcoplasmic reticulum of cardiomyocytes that plays a crucial role in excitation–contraction coupling. *RYR2* mutations disrupt calcium homeostasis in cardiomyocytes, leading to aberrant calcium release and alterations in cardiac action potential. These changes predispose individuals to the development of ventricular arrhythmias and sudden cardiac death, which are hallmark features of BrS. Furthermore, ryanodine receptor dysfunction leads to electrical remodeling of the heart, contributing to the arrhythmogenic substrate observed in BrS [70].

### 5.4. CALM

Calmodulin is a critical regulator of multiple cardiac ion channels involved in excitation–contraction coupling, including the ryanodine receptor (RyR2), L-type calcium channels (CaV1.2), and the cardiac sodium channel (NaV1.5). While CALM mutations have been primarily associated with early-onset syndromes such as catecholaminergic polymorphic ventricular tachycardia (CPVT) and long QT syndrome (LQTS) through dysregulation of RyR2 and CaV1.2, recent evidence suggests an expanded phenotypic spectrum [71]. The CALM2 G114R variant was found in a family with sudden deaths during sleep, a feature often seen in Brugada syndrome (BrS) and LQT3, both linked to NaV1.5 dysfunction. Functional studies showed that G114R and a related variant, G114W, greatly reduce calmodulin’s binding to the NaV1.5 IQ-domain—especially at low to moderate calcium levels—by over 50-fold. This likely disrupts the cardiac sodium current (INa) and promotes arrhythmias. Other CaM variants like N98S have milder effects, while non-arrhythmogenic variants such as I10T do not affect binding. However, CALM mutations more commonly cause severe early-onset arrhythmias like long QT syndrome (LQTS) and catecholaminergic polymorphic ventricular tachycardia (CPVT). Their role in BrS is less clear and may reflect variable or overlapping effects. More research is needed to define how CALM mutations contribute to BrS and arrhythmia risk.

## 6. Other Candidate Genes

### 6.1. TRPM4

The transient receptor potential melastatin member 4 (TRPM4) encodes a Ca^2+^-activated nonselective cation channel. Pathogenic TRPM4 variants have been reported in patients with inherited cardiac diseases, including conduction block and Brugada syndrome (BrS). Ozhathil et al. demonstrated that Trpm4 is expressed in atrial and ventricular myocytes, and interestingly, its deletion reduced peak Na^+^ currents in murine cardiac myocytes. While this suggests that TRPM4 expression may influence NaV1.5-mediated sodium current, the precise molecular mechanism linking TRPM4 to NaV1.5 regulation remains unclear and requires further investigation [72].

### 6.2. DLG1

A missense variant in DLG1, which encodes synapse-associated protein 97 (SAP97), was identified in a BrS patient. SAP97 is known to regulate cardiac ion channels and their localization. Studies using a murine model with inducible cardiac-specific SAP97 knockout revealed a downregulation of KV4.3 and Kir2.1 potassium currents, disrupted Kir2.1 localization, and an upregulation of NaV1.5 currents. These changes led to prolonged ventricular action potentials and increased arrhythmogenic events, reflected by ST and QT interval prolongation on ECG. Despite this, it remains uncertain whether the electrophysiological alterations observed in complete SAP97 knockout mice fully translate to human BrS patients carrying heterozygous DLG1 mutations, and further studies are needed to clarify this relationship [73].

### 6.3. Mitochondrial DNA (mtDNA)

Mitochondrial DNA (mtDNA) encodes essential subunits of the electron transport chain, which are critical for cellular energy production. Mutations in mtDNA, particularly those affecting mitochondrial tRNA genes, can impair the synthesis of respiratory chain proteins, potentially disrupting cardiomyocyte metabolism, calcium handling, and oxidative stress regulation. These disruptions may theoretically influence arrhythmia susceptibility [74]. However, while mitochondrial dysfunction has been implicated in a range of cardiac pathologies, there is currently no direct evidence establishing a causal relationship between mtDNA mutations and Brugada syndrome (BrS). Additionally, the suggestion that mtDNA variants might act as “modifiers” of BrS phenotype remains speculative and lacks supporting clinical or genetic data. Without more rigorous research and empirical evidence, the role of mtDNA in BrS pathogenesis remains unclear and requires further investigation before it can be considered a contributing factor [75].

### 6.4. RRAD

The *RRAD* gene, which encodes the Ras-related associated with diabetes (RRAD) protein, has been linked to BrS, resulting in changes in RRAD protein function and downstream signaling cascades. Disruption of RRAD regulation affects ion channel function and cardiac conduction, contributing to the clinical presentation of this syndrome. Induced pluripotent stem cell-derived cardiomyocytes with *RRAD* mutations show reduced action potential upstroke velocity, prolonged action potentials, increased early after-depolarization, decreased peak Na^+^ current amplitude, and altered actin distribution. These findings suggest that *RRAD* is a novel susceptibility gene for BrS [76].

GSTM3, a critical glutathione S-transferase, plays a key role in the cellular defense against oxidative stress. Experimental findings suggested that decreased GSTM3 levels during oxidative stress can lead to reduced cardiac sodium channel currents, potentially contributing to the development of BrS [77].

*DSG2* encodes a structural protein, desmoglein-2. A reduction in the sodium current density has been reported in *Dsg2* mutant mice [78]. *DSG2* mutations are linked to BrS, potentially through mechanisms involving alterations in cell junctions, increased arrhythmia susceptibility, and disruptions in desmosome structure and function, which affect cardiac electrical stability and contribute to the arrhythmogenic phenotype of the syndrome.

*LMNA*, a gene encoding lamin A/C, is implicated in the development of BrS. It is crucial for nuclear envelope integrity and function. Mutations in this gene can disrupt nuclear envelope structure, leading to altered gene expression and chromatin organization. These changes can dysregulate ion channel genes and contribute to arrhythmogenic phenotypes associated with BrS [79].

Telethonin (TCAP) is a crucial Z-disk protein responsible for maintaining cytoskeletal integrity and regulating signaling pathways within cardiomyocytes. TCAP modulates the α-subunit of the human cardiac sodium channel (hNa_V_1.5) via direct interactions [80]. Alterations in TCAP are associated with BrS, affecting cardiac ion channel functionality, interactions with contractile proteins, and cardiac electrophysiology. Two TCAP variants identified in patients with BrS induce loss-of-function modifications in the cardiac sodium channel (Na_V_1.5) within a cellular context.

*LRRC10* has been identified as a potential contributor to BrS because of its role in the regulation of cardiac ion channels and cardiomyocyte electrophysiology [81]. 

Disruption of *ZFHX3* gene expression may affect ion channel transcriptional regulation and cardiac repolarization [82], whereas *Xirp* [83] and *TMEM168* [84] have also been implicated in BrS, affecting ion channel function and membrane dynamics. Additionally, *TPM1* [85] and *CLCN1* [86] have been investigated for their potential involvement in this disorder, highlighting the complex genetic and molecular mechanisms underlying BrS.

## 7. Conclusions

This review summarizes candidate and well-established genes associated with BrS. These base mutations affect the function of the cardiac ion channels, intercellular communication, cytoskeletal structure, intracellular calcium ion balance, and other key physiological processes, leading to the onset of BrS. Recent advances in our understanding of the genotype–phenotype correlations have significantly enhanced our ability to predict arrhythmic events and refine risk stratification. The identification of specific genetic mutations, particularly loss-of-function mutations in SCN5A, has highlighted the importance of personalized risk profiling in guiding clinical decisions, such as the implantation of implantable cardioverter–defibrillators (ICDs). These genotype-specific risk profiles are crucial for determining which patients are at the highest risk for sudden cardiac death (SCD), and they help reduce the unnecessary implantation of ICDs in lower-risk individuals. Furthermore, the growing insight into the molecular mechanisms of BrS has opened up promising therapeutic avenues, particularly gene-targeted approaches such as gene therapy and allele-specific silencing. These innovative strategies aim to correct or mitigate the effects of pathogenic mutations, offering the potential for more definitive treatments. Gene editing techniques like CRISPR/Cas9 could, in theory, restore normal sodium channel function in cardiomyocytes, while allele-specific silencing could selectively target pathogenic alleles, reducing arrhythmic risk without affecting normal gene function. While still in the preclinical stages, these approaches hold promise for future clinical application. Next-generation sequencing (NGS) has revolutionized genetic screening for BrS, enabling the identification of a wider array of genetic variants associated with the condition. This technology has proven essential for detecting rare or novel mutations that may be missed by traditional genetic testing methods. By providing a comprehensive genetic profile, NGS can improve our understanding of the genetic heterogeneity of BrS, facilitate more accurate risk stratification, and enable early detection of at-risk individuals. This early identification can lead to preventive interventions such as lifestyle changes, medication adjustments, or timely ICD implantation, ultimately reducing the risk of sudden cardiac death.

However, despite these advancements, knowledge of the genetic and molecular foundations of BrS remains incomplete. Continued research into the pathophysiological mechanisms of BrS is essential for refining diagnostic strategies, enhancing risk assessment, and developing targeted therapies. As we gain a deeper understanding of BrS, the development of personalized diagnostic and treatment approaches will be key in improving outcomes for patients with this rare and often life-threatening disease. 

## Figures and Tables

**Table 1:** Genetic mutations and their impact on Brugada syndrome (BrS).

GENE	PROTEIN	MECHANISM	IMPACT ON BRS
SCN5A	NaV1.5	Na^+^ channel	Primary cause of BrS
SCN10A	NaV1.8	Na^+^ channel	Reduces INa
SCN1B	β1/β1b subunits	Na^+^ channel	Reduces INa
SCN2B	β2 subunit	Na^+^ channel	Reduces INa
SCN3B	β3 subunit	Na^+^ channel	Reduces INa
GPD1L	GPD1L	Na^+^ channel	Reduces INa
PKP2	Plakophilin-2	Na^+^ channel	Reduces INa
HEY2	Hey2	Transcription factor	Disrupts NaV1.5 expression
ANK2	Ankyrin-B	Na^+^ channel	Reduces INa
FGF12	FHF-1	Na^+^ channel	Reduces INa
RANGRF	MOG1	Na^+^ channel	Reduces INa
SLMAP	SLMAP	Na^+^ channel	Reduces INa
KCNE3	MiRP2	K^+^ channel	Increases Ito
KCND3	KV4.3	K^+^ channel	Increases Ito
KCNJ8	Kir6.1	K^+^ channel	Increases KATP current
KCNH2	hERG	K^+^ channel	Increases IKr
HCN4	HCN4	Na^+^/K^+^ channel	Reduces If current
ABCC9	SUR2A	K^+^ channel	Increases IK-ATP current
SEMA3A	Semaphorin 3A	K^+^ channel	Reduces Ito
CACNA1C	Cav1.2	Ca^2+^ channel	Reduces ICa
CASQ2	Calsequestrin 2	Ca^2+^ handling	Reduces ICa
RYR2	Ryanodine receptor 2	Ca^2+^ release	Reduces ICa
CALM	Calmodulin	Ca^2+^ binding	Reduces ICa
TRPM4	TRPM4	Ca^2+^-activated cation channel	Increases K^+^ current
DLG1	SAP97	Regulatory protein	Affects ion channel localization
MTDNA	Mitochondrial DNA	Mitochondrial function	Mitochondrial dysfunction
RRAD	RRAD	Signaling protein	Affects ion channel function
GSTM3	GSTM3	Oxidative stress	Reduces GSTM3 levels
DSG2	Desmoglein-2	Cell junction	Reduces sodium current
LMNA	Lamin A/C	Nuclear envelope	Alters nuclear envelope
TCAP	Telethonin	Z-disk protein	Affects sodium channel
LRRC10	LRRC10	Regulatory protein	Affects ion channels
ZFHX3	ZFHX3	Transcription factor	Affects ion channels
XIRP	Xirp	Membrane protein	Affects ion channels
TMEM168	TMEM168	Contractile protein	Affects ion channels
TPM1	TPM1	Contractile protein	Affects contractile function

This table summarizes the key genes associated with Brugada syndrome (BrS) and their corresponding mechanisms. It includes genes encoding sodium, potassium, and calcium channels, as well as other regulatory proteins. The mutations in these genes either disrupt ion channel function, alter ion current levels (e.g., sodium, potassium, calcium), or affect cellular processes, leading to electrophysiological abnormalities that contribute to the development of BrS.

## Data Availability

The datasets generated or analyzed during the current study are available from the corresponding author upon reasonable request.
